# Special Issue “Raman Spectroscopy: A Spectroscopic ‘Swiss-Army Knife’”

**DOI:** 10.3390/molecules24152852

**Published:** 2019-08-06

**Authors:** Keith C. Gordon

**Affiliations:** Department of Chemistry, University of Otago, Dunedin 9001, New Zealand; keith.gordon@otago.ac.nz

This special issue highlights the astonishingly wide range of scientific studies that use Raman spectroscopy to provide insight. There are many ways to factorize the papers in this issue, for example, based on the research area, there are contributions in materials for energy, health research, environmental monitoring, food research, and fundamental studies. However, one can equally well categorize around the types of experiment used, for example, time-dependent studies, temperature-dependent, redox cycling, ultra-sensitive, small domain (less than a micron) analyses. I describe some of the papers within the context of the research area while noting the experimental conditions that made the experiments possible.

In the field of new materials, the behavior of perovskites is the subject of a study from Yukio Furukawa’s group, their paper entitled Temperature-Dependent Evolution of Raman Spectra of Methylammonium Lead Halide Perovskites, CH_3_NH_3_PbX_3_ (X = I, Br) [1] describes the use of variable temperature Raman spectroscopy measurements to observe crystal changes form orthorhombic through tetragonal phases to the room temperature cubic phase. These changes can be monitored using bandwidth and Raman shifts with striking changes at the phase change temperatures. It demonstrates the potency of Raman spectroscopy as a method of understanding these important solar cell materials. In a study that highlights the versatility of Raman spectroscopy in the time-domain, the dynamics of charge-carriers in bulk heterojunction solar cell materials is described by Takaya et al. in Direct Observation of Structure and Dynamics of Photogenerated Charge Carriers in Poly(3-hexylthiophene) Films by Femtosecond Time-Resolved Near-IR Inverse Raman Spectroscopy [2]. In this remarkable study, the femto- to pico-second (ps) dynamics of charge carriers in regioregular poly(3-hexylthiophene) (P3HT):[6,6]-phenyl-C_61_-butyric acid methyl ester (PCBM) blend films and pristine P3HT films are investigated. In these studies, polarons (the key charge carriers in bulk heterojunction solar cells) are seen to be formed via two different pathways in under 10 ps. The nature of phase transitions in Li-ion batteries is the subject of a study by Noda and Jung entitled Investigation of the Phase Transition Mechanism in LiFePO_4_ Cathode Using In Situ Raman Spectroscopy and 2D Correlation Spectroscopy during Initial Cycle [3]. This study highlights the use of 2D correlation spectroscopy, this is an elegant and insightful method of observing subtle spectral changes (both synchronous and asynchronous) in systems undergoing perturbation. In this case, charge and discharge is the perturbation and it is shown that residual species are present through the cycling that cannot be easily seen in the Raman spectra without 2D correlation analysis. The cycling of redox is the topic of the paper by Krige et al. On-Line Raman Spectroscopic Study of Cytochromes’ Redox State of Biofilms in Microbial Fuel Cells [4]. Using biofilms, the group were able to monitor the oxidation state of cytochrome oxidase by control of the poise potential in a stack-microbial fuel cell. Redox changes at a molecular level are the subject of the study by Zedler et al. (Resonance Raman Spectro-Electrochemistry to Illuminate Photo-Induced Molecular Reaction Pathways [5]) in which they use resonance Raman spectroelectrochemistry to understand the flow of charge in a molecular species [RuII(tbtpy)_2_]^2+^ (tbtpy = tri-tert-butyl-2,2′:6′,2″-terpyridine). Using sophisticated density functional theory calculations to model the resonance Raman spectra of both the ground state complex and its redox products, the authors show that the doubly reduced complex (an important intermediate in photocatalysis) has each of the reducing electrons on each of the terpyridine ligands.

In the area of environmental monitoring and food research, Toganaccini et al. show in their paper Surface Enhanced Raman Spectroscopy (SERS) for In-Field Detection of Pesticides: A Test on Dimethoate Residues in Water and on Olive Leaves [6] that is it possible to use surface-enhanced Raman spectroscopy to detect organophosphate insecticides on leaves of olive plants. Critically, they show that this can be done in-field using portable Raman spectrometers and SERS substrates. This highlights that ability to measure very low concentrations of analyte (10^−7^ M) via the SERS effect. Food quality is the subject of the study by Broadhurst et al. Continuous Gradient Temperature Raman Spectroscopy of Fish Oils Provides Detailed Vibrational Analysis and Rapid, Nondestructive Graphical Product Authentication [7]. In this work, a group of fish oils from differing manufacturers and subject to differing processing are readily differentiated providing a rapid method of assuring quality and product authentication.

In the area of health, surface-enhanced Raman spectroscopy was utilized by Tien et al. in Diagnosis of Bacterial Pathogens in the Urine of Urinary-Tract-Infection Patients Using Surface-Enhanced Raman Spectroscopy [8]. In this study, a SERS chip is used to detect pathogens in urine samples within a clinic. Using multivariate analysis, it is possible to identify bacteria such as *E. coli* and *Staphylococcus aureus* with a test that takes only a few minutes. More complex samples in which there are many types of bacteria (so-called mixed flora) can also be categorized, but detailed identification remains challenging, as the Raman data set is inchoate. Multivariate analysis is combined with Raman microscopy in the study of bone mineral density (BMD) in Raman Spectroscopic Analysis to Detect Reduced Bone Quality after Sciatic Neurectomy in Mice [9] by Ishimaru et al. Their study uses the spectra of bone containing the phosphate carbonate and protein signatures to map the disabling effects on bone structure in neurectomized mice versus normal. The carbonate to phosphate ratio is enhanced in the neurectomized mice and this is also seen in principal component analysis scores plots. The work is supplemented with micro-CT scanning and BMD measurement. Single-cell analysis can be done using Raman spectroscopy and this is highlighted in the study, Anti-Cancer Drug Sensitivity Assay with Quantitative Heterogeneity Testing Using Single-Cell Raman Spectroscopy [10] by Zhang et al. Using confocal Raman microscopy, they use Raman spectral intensities to monitor the effect of drugs on single cells. The identification of dietary supplements by a simple drying and analysis method is highlighted by Li et al. in Rapid Detection of Six Glucocorticoids Added Illegally to Dietary Supplements by Combining TLC with Spot-Concentrated Raman Scattering [11].

Finally, the special issue contains two review articles that are focused on the use of Raman spectroscopy to understand some very new materials (two-dimensional systems) and some very old materials (cellulose and lignocellulose). Dong et al. in Raman Characterization on Two-Dimensional Materials-Based Thermoelectricity [12] describe how using variable temperature Raman studies, it is possible to derive figures of merit for thermoelectric performance in a variety of 2D materials. Agarwal reviews the use of Raman spectroscopy in cellulosic materials in Analysis of Cellulose and Lignocellulose Materials by Raman Spectroscopy: A Review of the Current Status [13] including discussion of stimulated Raman scattering and coherent anti-stokes Raman scattering as methods of evaluating sub-cellular structures in raw corn stovers.
